# Assessing the contraceptive supply environment in Kinshasa, DRC: trend data from PMA2020

**DOI:** 10.1093/heapol/czx134

**Published:** 2017-11-10

**Authors:** S Babazadeh, S Lea, P Kayembe, P Akilimali, L Eitmann, P Anglewicz, J Bertrand

**Affiliations:** 1 Global Health Management and Policy, Tulane University School of Public Health and Tropical Medicine, 1440 Canal St, Suite 1900, New Orleans, LA 70112, USA and; 2 Kinshasa School of Public Health, Kinshasa, Democratic Republic of the Congo

**Keywords:** Family planning, contraception, assessment, developing countries, evaluation, health services, programmes, survey

## Abstract

Performance Monitoring and Accountability 2020 (PMA2020) is a population-based and facility-based survey program conducted in 11 countries to track contraceptive use dynamics and the supply environment. Annual data collection provides trend data unavailable from any other source. Two-stage cluster sampling was used to select 58 enumeration areas in Kinshasa; data were collected in 2014, 2015 and 2016 from three to six service delivery points (SDPs) per EA. Of the 228–248 SDPs surveyed each year, only two-thirds reported to offer family planning (FP) services. Of those reporting to offer FP, one-fifth or more did not do so on the day of the survey. As of 2016, only one-half of SDPs offering FP had at least three methods available, a proxy for contraceptive choice; only one in five had at least five methods. Long-acting reversible contraceptives, including implants and IUDs, were less widely offered and more often stocked out than resupply methods, including condoms, pills and injectables. Contraceptive stockouts were rampant: in 2016, over a quarter of the SDPs experienced stockouts of all methods (except condoms) in the previous 3 months, and two of the three most widely used methods—implants and injectables—were also the most likely to be stocked out. The findings documented the inconsistency in pricing of methods across facilities; moreover, less than one quarter of SDPs posted prices. Patterns in the contraceptive supply environment remained relatively unchanged between 2014 and 2016. The PMA2020 SDP module provides timely, actionable information to the DRC government, FP implementing organizations and donors involved in FP service delivery in Kinshasa, DRC. Yet the value of this information will be determined by the ability of the local FP stakeholders to use it in bringing the needed improvements identified by this survey to the contraceptive supply environment.

Key Messages

- PMA2020 represents an underutilized resource for tracking the FP supply environment in developing countries.
- Repeat facility-based surveys in Kinshasa, DRC, document widespread contraceptive stockouts and lack of transparency in pricing.
- The value of these data depends on the actions that the government, implementing agencies and donors take to address these shortcomings.

## Introduction

With one of the world’s largest (83 million) and most rapidly growing populations (at 3.1% per annum), the government of the Democratic Republic of the Congo (DRC) is focused intently on future population growth and its implications. In its national strategic plan for family planning (FP), the DRC established the objective of increasing the modern contraceptive prevalence rate (MCPR) for all women of reproductive age to 19.0% by 2020 (Ministry of Health Democratic Republic of the Congo 2014). As of 2013–14, MCPR was 7.8% among married women, and 8.1% among all women for the country as a whole (Ministère du Plan et Suivi de la Mise en œuvre de la Révolution de la Modernité et al. 2014).

Kinshasa represents a priority focus for FP activities in the DRC because (1) it is the capital city, (2) it constitutes 14% of the total population of the country, (3) behaviours adopted in Kinshasa are likely to diffuse to other parts of the country and (4) it serves as a ‘test of concept’ for intervention strategies that can then be replicated elsewhere in the country, as reported in Binanga and Bertrand (2016). Thus, developing an FP program characterized by high access and quality is a priority for the government, donors, and FP implementing agencies working in Kinshasa. MCPR among women married or in union in Kinshasa has increased over the past 4 years, from 18.5% (in 2013) to 23.8% in 2016 (Performance Monitoring and Accounability 2020 [PMA2020] 2014, 2015, 2016a). Yet by international standards, it remains very low.

MCPR is a function of both supply (what is available) and demand (what the population wants) in terms of contraception. A new survey mechanism ‘Performance Monitoring and Accountability 2020’ (PMA2020) collects population-based data that captures contraceptive use dynamics and facility-based data that measure the supply environment for FP services. Three innovative features of PMA2020 are: (1) data collection using smartphone technology, (2) use of interviewers that reside in their enumeration area (EA) (thus referred to as resident enumerators or ‘REs’) and (3) annual data collection for both the population- and facility-based surveys. The name ‘PMA’ relates to the FP2020 goal of expanding access to modern contraception information, services and supplies—to 120 million additional women and girls in the world’s poorest countries by 2020 (Brown *et al.* 2014). PMA2020 is now active in 11 countries, 9 in Sub-Saharan Africa and 2 in Asia (PMA2020 2016b).

The PMA2020 data are particularly relevant in the context of Kinshasa, DRC, where a number of government agencies and non-government organizations (NGOs) are working to increase modern contraceptive prevalence by increasing access to quality FP services (‘supply’) and heightening awareness and motivation for using contraception (‘demand’). This research illustrates the value of the PMA2020 service delivery point (SDP) module in measuring key aspects of the supply environment over time, including: availability of FP services in healthcare facilities, choice (number of contraceptive methods at a given facility), frequency of contraceptive stockouts and cost of contraception to the clients.

The PMA2020 data offer a rare opportunity to track trends in FP services in Kinshasa. Other facility-based surveys that preceded PMA2020 in measuring the supply environment for FP in developing countries include the Situation Analysis (Miller *et al.* 1997) and the Service Provision Assessment (SPA) of the Demographic and Health Surveys (DHS) (The Demographic and Health Surveys Program [DHS Program] 2017). However, in contrast to these one-off or occasional assessments, the PMA2020 SDP survey tracks the supply environment every 6–12 months, allowing for trends to be identified in near real time. This opportunity to track changes in FP service delivery in an environment where such services are critical to national interests and are also rapidly evolving represents a major contribution to FP programming. To our knowledge this is the first article in the peer-reviewed literature to analyse trends from the SDP module of PMA2020 using three rounds of data collection.

## Methodology

### Study design and sampling

Despite the clear importance of FP SDPs in providing and measuring the supply of contraception, SDP data are generally lacking in settings with rapidly-growing populations. While surveys such as DHS regularly collect data on contraceptive use from women of reproductive ages (thereby measuring demand and use), data on contraceptive supply from SDPs is collected far more infrequently, and only in a sub-set of DHS countries (through the SPA) (DHS Program 2017). Furthermore, when facility-based data have been collected, it is rare to have repeated cross-sectional data to measure trends in contraceptive supply.

PMA2020 conducts three surveys in all countries: female, household and SDP. The PMA2020 sampling approach was designed to obtain a sample that is representative of Kinshasa. PMA2020 used two-stage cluster sampling, in which the study first randomly selected 58 census EAs within Kinshasa (out of a total of 335). For the female and household surveys, PMA2020 first conducted a listing of all households in these EAs, and randomly selected 33 households within each EA. All resident women of reproductive ages (15–49 years) within the household were selected for interview. For women who consented to be interviewed, the PMA2020 female and household surveys included basic demographic information, fertility, contraceptive use and other related measures. Data were also collected in Kongo Central for Round 4 but are not relevant to this analysis.

The SDP survey attempted to collect data from a maximum of six SDPs per EA: up to three public (government) and three private. The sampling approach differed between public and private SDPs. For private facilities, the REs first conducted a listing of all private facilities within the EA. Private health facilities included faith-based SDPs, pharmacies and chemists, private clinics and other. From the full list of private facilities, the survey supervisor randomly selected three for the RE to interview.

For public SDPs, the survey team obtained from government health authorities a list of all public facilities designed to serve each EA in the sample, including lowest level health clinics, intermediate level hospitals/health centres and tertiary hospitals. For each EA, the tertiary hospital was automatically included; there is one tertiary hospital in DRC, so it is shared by all EAs; all secondary hospitals were also included, even if not in the EA itself, but as long as it served the EA. If the EA had more than one of the lowest level facilities that served its residents, one was randomly selected for interview. Due to the fact that EAs shared public health facilities, not all EAs had six total SDPs in the sample. Nearly all EAs had three or more private SDPs, however. The total number of SDPs per EA ranged between 3 and 6.

The data for this analysis were collected between 2014 and 2016 in the city of Kinshasa, DRC. These surveys are referred to as Rounds 2, 3 and 4 in other publications (PMA2020 2014, 2015, 2016a). In addition, Table 1 includes 2016 data from the female survey in order to compare availability of contraceptive products to the preferred source of supply among users.

**Table 1.** Availability of most widely used contraceptive methods by type of SDP^a^ compared with source of methods among contraceptive users, Kinshasa, 2016

	Condom		Implant		Pill		Injectable		IUD	
	Available at SDP^a^	Source among users^b^	Available at SDP^a^	Source among users^b^	Available at SDP^a^	Source among users^b^	Available at SDP^a^	Source among users^b^	Available at SDP^a^	Source among users^b^
Percent of modern users that used this method	29.3		28.4		17.4		16.6		1.7	
Hospital (*n* = 19)	84.2	3.6	73.7	46.7	89.5	6.9	84.2	21.6	68.4	50.0
Health centre (*n* = 41)	61.0	1.3	56.1	35.0	65.9	3.9	56.1	47.3	43.9	16.7
Pharmacy (*n* = 80)	88.7	73.5	0.0	0.0	45.0	79.4	21.3	8.1	0.0	0.0
Clinic (*n* = 2)	100.0	1.0	50.0	13.3	100.0	1.0	50.0	2.7	100.0	33.3
Other (*n* = 4)	50.0	17.2	100.0	4.2	75.0	7.8	75.0	20.3	100.0	0.0
Missing or DK	–	3.3	–	0.8	–	1.0	–	0.0	–	0.0

a Data from the SDP survey in 2016

b Data from the female survey (women 15–49 years old) in 2016

### Measurement

Primary measures of interest included the provision of FP services. Whether or not an SDP offered FP services was determined by self-report from the SDP in response to the survey question, ‘Do you usually offer FP services/products?’ Specific contraceptive methods were considered to be ‘available’ if the SDP reported offering this method and if it was in stock on the day of the survey. Due to an evolution in the recommendations for measuring stockouts, the reference period (past 3 months vs past 6 months) changed over the 3-year period of PMA2020 data collection in Kinshasa, resulting in non-comparable results for 2014 as compared with 2015 and 2016. Stockouts were considered to occur if an SDP reported to offer a given method but it was not in stock on the day of the survey or was unavailable at any point in the reference period.

SDP assessments fit within the Donabedian quality of care framework that includes structure, process and outcome (Donabedian 1979). The PMA2020 SDP survey captures capture one dimension of quality of care: readiness to provide services (which corresponds to ‘structure’). In contrast, they do not necessarily capture the quality of the actual service delivered to clients. The PMA2020 SDP module measures several aspects of readiness (e.g. number of methods provided and available, trained staff, number of days offering services, information given to clients and integration of FP in other programs). However, it does not include on-site third-party observations and client interviews that would measure the technical competence of service providers or the nature of the interpersonal interaction between providers and clients (Leisher *et al.* 2016; Tumlinson 2016). As such, some would call this particular survey a ‘facility audit’ or a ‘readiness survey’ rather than a facility-based survey. A facility audit is part of a facility-based survey, but a facility-based survey is not exclusively the audit.

### Data collection

The three rounds of data collection took place during the following time periods: Round 2, August–September 2014; Round 3, May–June 2015; and Round 4, October 2015–January 2016 (labelled herein as 2016).

PMA2020 also included Round 1, but the SDP module was not included in the first round. Although the start date for each round of data collected took place at 6–9 months intervals, we labelled each round of data by the three successive years in which some data were collected to give a temporal context to the results.

Upon arrival at each facility, the enumerator or supervisor asked to speak with the person in charge. Using a consent script, the enumerator/supervisor then asked the person in charge for consent to participate in the study. No information was collected that would identify the name of the person in charge. Once consent was received, the enumerator/supervisor administered a detailed survey using mobile smartphones programmed with OpenDataKit (ODK) software. The questionnaire included information on number of years the SDP has been open, number of days FP services are offered per week, presence of staff trained in FP, methods of contraceptives available on the day of the survey, recent stockouts for contraceptive methods and cost of FP services. The mobile smartphones were also used to photograph and identify the geographic coordinates for each SDP.

### Data analysis

In our analysis, we tabulated SDP characteristics by year for all SDPs and those offering FP. The remainder of the analysis was based on facilities that reported providing FP services. We conducted chi-squared tests of differences by year on two variables: availability of contraceptives and stockouts. A key explanatory variable in the bivariate analyses was facility type, defined as hospital, health centre, health clinic, health post, pharmacy and other. Although facilities were classified as public (governmental) or private, we did not use this as an explanatory variable for two reasons: (1) only a fraction of the total facilities were public (ranging from 8 to 14% over the three surveys), and (2) the distinction between public and private sector facilities is often blurred, for example, when an international NGO provides training and commodities to a government facility (Kayembe *et al.* 2015). Data from all three SDP surveys were analysed using Stata 14 software.

This study received approval to collect female, household and SDP data from Institutional Review Boards at Johns Hopkins University, Tulane University and the University of Kinshasa.

## Results

The total number of SDPs surveyed in each of the 3 years (2014–16) was 248, 248 and 228. Of the six types of SDPs (health centres, pharmacies, hospitals, health clinics, health posts and other), health centres and pharmacies made up over 83% of SDPs included in all 3 years of the survey. Health centres accounted for 47.6 and 49.2% in of the total sample in 2014 and 2015, but dropped to 40.8% in 2016. In contrast, pharmacies made up 39.5 and 34.3% of the total sample in 2014 and 2015, and increased to 43.9% in 2016. Hospitals accounted for 10% or less of all SDPs in each survey, and the remaining SDP types (health clinic, health post and other) each represented <5% of SDPs; see Table 2.

**Table 2.** Characteristics of SDPs surveyed in Kinshasa, DRC: PMA2020 2014, 2015 and 2016

Among all SDPs surveyed	2014 (*N* = 248)	2015 (*N* = 248)	2016 (*N* = 228)
Types of SDPs surveyed	**%**	**%**	**%**
Hospital	7.7	9.7	10.1
Health centre	47.6	49.2	40.8
Health clinic	2.4	0.8	1.3
Health post	1.2	1.6	1.3
Pharmacy	39.5	34.3	43.9
Other	1.6	4.4	2.6
SDPs that offered FP services^b^	63.7	67.7	64.0
Hospital	84.2	83.3	82.6
Health centre	45.8	49.2	44.1
Health clinic	100.0	^a^	^a^
Health post	^a^	^a^	^a^
Pharmacy	81.6	91.8	80.0
Other	^a^	63.6	66.7
**Among SDPs that offered FP services**	**2014 (*n* = 158)**	**2015 (*n* = 168)**	**2016 (*n* = 146)**
Type of SDP			
Hospital	10.1	11.9	13.0
Health centre	34.2	35.7	28.1
Health clinic	3.8	1.0	1.4
Health post	1.0	1.2	0
Pharmacy	50.6	46.4	54.8
Other	1.0	4.2	2.7
Managing authority of SDP			
Private	54.4	59.5	59.6
NGO	18.4	12.5	11.6
Faith-Based	15.8	12.5	8.9
Government	10.8	14.9	19.2
Other	0.6	0.6	0.7
Number of trained staff at SDP			
0	21.3	33.6	29.4
1–5	44.1	32.2	43.7
6–10	19.1	19.9	11.1
11 or more	15.4	14.4	15.9
Median # clinical staff	2.0	3.0	2.0
Mean # years FP services have been offered	6.1	5.6	4.8
Median # years FP services have been offered	2.8	2.3	1.8
Number of days per week SDP offers FP services			
1–2 days	13.9	11.3	11.0
3–5 days	12.0	13.1	11.6
6 days	32.9	29.8	30.8
7 days	39.2	43.5	15.2
Do not know, no response or 0 days	1.9	2.4	1.4
Mean number of days FP services are offered	4.2	4.5	5.0
Median number of days FP services are offered	6.0	6.0	6.0

a We do not provide percentages for offering services when SDP *n*≤5.

b ‘Offer FP services’ is defined as those SDPs that responded ‘yes’ to the question, ‘Do you usually offer FP services/product’

Among all SDPs surveyed, 63.7% (2014), 67.7% (2015) and 64.0% (2016) reported to offer FP services; however, of those SDPs that reported to offer FP services, close to one in five (18.5–25.9%) did not offer FP services on the day of the survey; see Table 2. The percentage of SDPs that report NOT offering FP services reflects the magnitude of opportunity for introducing FP in existing health facilities. The percentage of SDPs that report to offer FP but have no contraceptives in stock represents a failure of management to provide contraceptives where there is an organizational willingness to do so.

The rest of the analysis is based on those SDPs that reported to offer FP services, whether or not they were available on the day of the survey.

### Characteristics of facilities offering FP services

In all 3 years of the survey, the managing authority for the vast majority of SDPs offering FP was private (>80%), including NGO and faith-based organizations, whereas government-managed SDPs constituted <20% (Table 2). In all 3 years, 99–100% of pharmacies were private; data not shown.

The median number of clinical staff (including doctors, medical officers, nurses, midwives, nursing assistants/aides and pharmacists) at these SDPs was 2–3 in each year. The median number of staff per facility varied greatly by type of facility (with hospitals having two to three times as many staff as health centres or clinics); pharmacies and health posts averaged less than two staff. However, this question was not specific to staff that provided FP services or products. In 2016, SDPs had been in operation on average (median) for 3 years, and they operated on average (median) 6 days a week; see Table 2.

### Availability and choice of contraceptive methods

We measured availability of contraceptive methods in three ways: the percentage of SDPs offering FP services that had each specific contraceptive method available, those with at least three methods available, and those with at least five methods available. These latter two variables evaluate a key principle in international FP programs: ensuring that clients have a choice of methods.

**Figure 1.**
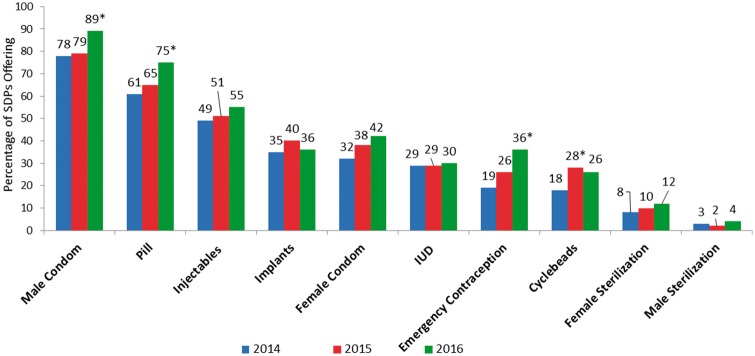
Availability of specific contraceptive methods at SDPs, PMA2020 Kinshasa, 2014–16. *Chi-squared test of difference with 2014 is significant at *P*<0.05

PMA2020 yielded the percentage of SDPs that had each specific contraceptive method available by year; see Figure 1. The methods most readily available were male condoms, the pill, and injectables, with the percentage of SDPs with each method available increasing all 3 years*.* SDPs were more likely to have short-term reversible methods—including male condoms, pills and injectables—than long-acting reversible contraceptives (LARCs), such as implants and IUDs. In contrast, female and male sterilization services were available at < 8 and 3% of SDPs, respectively. The two methods with the largest percentage point increase for availability between 2014 and 2016 were emergency contraception and pills (difference by year is statistically significant at *P* < 0.05), whereas the smallest increases over the same period were seen in LARCs, specifically implants and IUDs.

One measure of quality of FP services is having a range of contraceptive methods available, which PMA2020 captures as ‘at least three methods’ and ‘at least five methods’ (PMA2020 2014, 2015, 2016a). The percentage of SDPs with at least three methods increased over the three surveys, from 43.0% (2014) to 46.4% (2015) to 53.4% (2016); see Table 3. In other words, close to half the facilities that reported offering FP methods did not adhere to the principle of offering a range of methods (with three considered to be the bare minimum). Hospitals were more likely to have at least three contraceptive methods than other SDPs, and this percentage jumped from 68.8% (2014) to 89.5% (2016). Health centres followed close behind in 2014 at 61.1% but dropped to 48.3% in 2015 before increasing to 70.7% in 2016. One-third or less of the pharmacies had at least three methods available.

**Table 3.** Percentage of SDPs that offered family planning in Kinshasa with three or more and five or more modern contraceptive methods available^b^, by year

	2014		2015		2016	
	*n*	%	*n*	%	*n*	%
3 or more methods available	158	43.0	168	46.4	146	53.4
Hospital	16	68.8	20	85.0	19	89.5
Health centre	54	61.1	60	48.3	41	70.7
Health clinic	6	16.7	2	^a^	3	^a^
Health post	1	^a^	4	^a^	3	^a^
Pharmacy	80	28.8	78	34.6	80	32.5
Other	1	^a^	7	42.9	4	^a^
5 or more methods available	158	20.3	168	16.9	146	20.6
Hospital	16	56.3	20	50.0	19	84.2
Health centre	54	37.0	60	28.3	41	53.7
Health clinic	6	16.7	2	^a^	3	^a^
Health post	1	^a^	4	^a^	3	^a^
Pharmacy	80	2.5	78	1.3	80	6.3
Other	1	^a^	7	27.3	4	^a^

a We do not provide percentages for offering services when SDP *n*≤5

b Available is defined as being in stock and observed on the day of the survey for implants, IUDs, injectables (Depo Provera and Sayana Press), pills, male condoms, female condoms, emergency contraceptives, diaphragms, foam and beads; and as being provided by the facility for female sterilization and male sterilization

A lower percentage of SDPs—about one in five—had at least five methods available, meaning a larger range of methods. Again, hospitals were most likely to have five or more methods available, with percentages dropping from 56.3 to 50.0% before increasing to 84.2% over the three surveys. Health centres followed the same pattern, dropping from 37.0 to 28.3% before increasing to 53.7% in 2016. Pharmacies trailed far behind, with only 1.3–6.3% offering at least five methods in any of the three survey years. Because the samples of health clinics, health posts and ‘others’ were below five in each survey, their percentages are not included in Table 3.

This article focuses primarily on the SDP survey, yet PMA2020 also yields data from the same EAs for women of reproductive age. Specifically, it allowed us to compare the availability of methods with the source from which actual users of contraception obtain their methods; see columns 1 and 2, respectively, of Table 1.

The type of facility where users reported obtaining their contraception differs markedly by method, shown in Table 1. The large majority of condom users (73.5%) and pill users (79.4%) got their supplies from a pharmacy; injectable users were most likely to obtain the method from health centres (47.3%). In contrast, women using LARCs were most likely to cite a hospital as their source: implants (46.7%), IUDs (50.0%).

These patterns are consistent with expectations. Yet from the perspective of a program manager, it is notable that over 60% of hospitals and health centres carried condoms and pills, yet <7% of condom or pill users got their method from hospitals or health centres.

### Stockouts of contraceptive commodities

**Figure 2.**
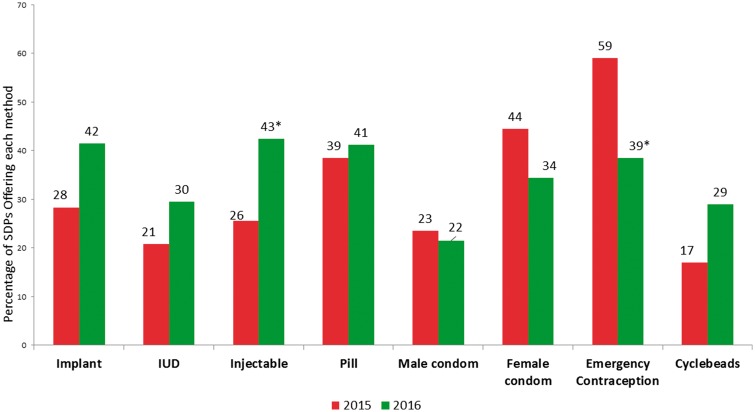
Percentages of SDPs that experienced stockouts in past 3 months, by method, PMA2020 Kinshasa, 2015 and 2016. *Notes*: *Chi-squared test of difference between 2015 and 2016 is significant at *P*<0.05; the denominator in these percentages is the number of SDPs that report offering the specific method at the time of the survey

Stockouts were measured for the most recent 6-month period in 2014 as well as for the most recent 3-month period in 2015 and 2016 (as explained in the methodology). At least 17% of SDPs reported stockouts of every method of contraception in 2015 and 2016; see Figure 2. The percentage of SDPs reporting stockouts increased for all methods between the two surveys, except for male condoms, female condoms and emergency contraception. Chi-squared tests of differences by year showed a statistically significant decline in stockouts of emergency contraception and a significant increase in stockouts of injectables (*P* < 0.05). In 2016, more than one-quarter of SDPs reported experiencing stockouts of all types of contraceptive commodities in the previous 3 months, except male condoms (21.5%). The methods for which the highest percentage of SDPs reported stockouts in 2016 were injectables (42.5%), implants (41.5%) and emergency contraception (38.5%).

### Payment for contraceptives

As of 2013 the DRC ranked next to last on the Human Development Index; 74% of the population of the DRC was living in multidimensional poverty (United Nations Development Programme 2013). Thus, the cost of contraceptives, however minimal by the standards of developed settings, can potentially influence a woman’s decision to adopt a method or not. Moreover, in many facilities the cost of the method is only one component of the cost of obtaining a method; a client may also have to purchase a *fiche de consultation*, a client card that covers the cost of the visit, as well as supplies required to apply the method, such as cotton and antiseptic in the case of an injection or insertion of the implant. The National Program of Reproductive Health (PNSR) has established a list of prices that all facilities in Kinshasa are expected to follow, but anecdotal evidence suggests that prices are by no means standardized across facilities in the city. The SDP module asked the price of each method provided by the SDP. It also assessed whether a list of prices was posted in the facility, as a means of informing clients of the price they should expect to pay and thus reinforcing the concept of a standardized price for each method.

The percentage of SDPs that report charging for contraceptive methods was 81.0% in 2014 and 80.4% in 2015, dropping to 71.2% in 2016; see Table 4. Although pharmacies tended to be more likely than other SDPs to charge for methods, one anomalous finding was that the percentage of pharmacies that reported NOT charging for contraceptives increased from 15.4 to 30.0% from 2015 to 2016.

**Table 4.** Percentage of SDPs in Kinshasa offering FP services that charge a fee for FP services and that post their fees, by facility type^a^ and year

	2014		2015		2016	
	*N*	%	*n*	%	*n*	%
Fees charged for FP services	158	81.0	168	80.4	146	71.2
Hospital	16	68.8	20	85.0	19	63.2
Health centre	54	75.9	60	76.7	41	78.1
Health clinic	6	66.7	1	^a^	2	^a^
Health post	1	^a^	2	^a^	–	–
Pharmacy	80	87.5	78	84.6	80	70.0
Other	1	^a^	7	57.1	4	^a^
Fees for FP Services Posted^b^	128	12.5	135	14.1	104	24.0
Hospital	11	18.2	17	11.8	12	25.0
Health Center	41	31.7	46	19.6	32	34.4
Health Clinic	4	^a^	1	^a^	2	^a^
Health post	1	^a^	1	^a^	–	–
Pharmacy	70	1.4	66	12.1	56	16.1
Other	1	^a^	4	^a^	2	^a^

a We do not provide percentages for offering services when SDP *n* ≤ 5

b Both ‘yes, all fees are posted’ and ‘Yes, some, not all fees are posted’ are included

Posting the prices of contraceptives is still not a common practice in Kinshasa, although the percentage of SDPs posting prices increased from 12.5% in 2014 to 24.0% in 2016. In all years, health centres were more likely to post prices than hospitals; pharmacies were the least likely to post prices of contraceptives.

## Discussion

What do the PMA2020 SDP data tell us about the performance of FP service delivery in Kinshasa over the past 3 years (2014–16)? These data provide a rare opportunity to track the supply of contraceptives in an environment with rapid population growth.

Given the shortcomings they reflect in the supply environment, the findings of this study should inspire a call to action for the government, FP program managers and international donors. Approximately one-third of health facilities did not provide FP services, and among those reporting to offer FP services, one-fifth or more did not offer services on the day of the survey. As of 2016, only one-half of health facilities offering FP services had at least three methods available, and only one in five had at least five methods available. Hospitals, health centres and clinics did improve in this measure between 2014 and 2016, whereas pharmacies were the least likely to offer a range of contraception. Among all methods, LARCs—specifically implants and IUDs—were less readily available than supply methods, including condoms, pills and injectables. Contraceptive stockouts were rampant: one in 10 SDPs reported stockouts for every method in all 3 years. In 2016, over a quarter experienced stockouts of all methods except condoms in the previous 3 months, and two of the three most widely used methods—implants and injectables—were also the most likely to be stocked out. Posting of the price of contraceptives remains relatively rare, though improved since 2014. By 2016, only one in four SDPs posted prices at the site.

The findings also provide useful insight into other dynamics of the FP service environment. Although pharmacies carried fewer contraceptive methods on average than other health facilities, they remain the predominant source of supply for users of condoms and pills. Consistent with the principle of choice (a large range of methods), over 60% of hospitals and health centres carried condoms and pills; yet fewer than 7% of users of these two methods obtained them from hospitals or health centres. The practice of charging for FP services vs delivering them free of charge varied markedly across facilities in Kinshasa, in particular by type of facility. As of 2016, 71.2% of all SDPs required payment. Facilities that offer methods free of charge (to some or all clients) increase access to contraception; yet the lack of consistency in pricing—even within the same type of facility—creates uncertainty for clients. The problem is exacerbated by the absence of price posting in three-quarters of facilities.

This article identifies clear shortcomings in FP service delivery in Kinshasa. Yet these results must be viewed within the context of challenges that confront the delivery of any health service in the DRC: deteriorating infrastructure, shortage of qualified health personnel, low government salaries—when they are paid at all, overcrowded health facilities, absence of sanitary facilities, frequent power outages, shortage of computers and infrequent internet connectivity, among others. One must also consider factors beyond the control of the FP implementing agencies that manage these services. For example, in recent years the flow of donor-funded contraceptives into the country has been sporadic and inadequate to meet the need.

The task of addressing these challenges is further complicated by the structure of FP service delivery in Kinshasa. Whereas the PNSR is responsible for establishing norms and guidelines, the actual implementation of FP service delivery relies largely on a network of local and international NGO partner organizations. In Kinshasa alone, at least 10 different organizations are responsible for providing contraceptive supplies to the SDPs that they support (A Binanga 2016, personal communication). This system contrasts markedly to one that has a single entity responsible for resupplying SDPs with contraceptives, such as the Informed Push Model used in Senegal. After the implementation of the Informed Push Model in 140 public facilities in the Dakar region, stockout rates dropped to < 2% for the year (Daff *et al.* 2014).

The PMA2020 study design has several notable strengths, including data collected over three consecutive years, and a representative sample of the main types of health facilities. Also, the study reports on indicators of key importance to the FP community: method availability by type of contraceptive, availability of trained personnel, fees charged, posting of fees, and stockouts—the latter which has emerged in recent years as an increasingly important indicator to the international FP community (Reproductive Health Supplies Coalition 2015). Overall, the PMA 2020 study provides a comprehensive description of the FP supply environment in Kinshasa.

While PMA2020 improves upon previous efforts to measure SDP characteristics, there are some limitations to the study design. The geographic scope of PMA2020 within DRC is limited to only two provinces (out of 26). While nearly all measures were consistent across PMA2020 surveys, there were some notable changes: in measuring stockouts, the reference period changed over the 3-year period of PMA2020 data collection in Kinshasa (from the past 3 months to the past 6 months), resulting in non-comparable results for 2014 as compared with 2015 and 2016; for this reason, we reported on the two most recent years in the analysis. Other important SDP measures are not included in the PMA2020 survey. Stockouts are considered to occur if an SDP reports to offer a given method, but it is not in stock on the day of the survey or has been unavailable at any point in the previous 3 months. Yet the survey does not include a measure of the percentage of SDPs that could or should be providing the method but do not, which is an important but separate indicator for FP services at SDPs.

It is useful to weigh the value of PMA2020 for measuring the FP supply environment in comparison to similar research conducted in Kinshasa. In 2012, Kayembe *et al.* attempted to identify the universe of FP SDPs; the team repeated the data collection in 2013. The survey showed an increase in the number of SDPs offering FP services and the percentage that showed ‘readiness’ to deliver FP services; yet it reflected the same lack of contraceptive availability as the later PMA2020 surveys (Kayembe *et al.* 2015). One advantage of conducting a survey of the universe of facilities was the ability to provide systematic feedback to implementing partners on performance across the sites that each organization supported for FP service delivery. However, the questionnaire for this study was limited to only a few variables.

In 2016, Population Services International conducted the FPwatch survey in Kinshasa (among other locations) to obtain high-quality information on modern contraceptive availability, price and relative market share, as well as contraceptive service availability and readiness through outlet surveys (Population Services International and FPWATCH 2016). It provided a full census of all outlets providing contraceptive methods within selected EAs, a full audit of all available contraceptive commodities, and a provider interview on contraceptive services—among randomly selected health areas in Kinshasa. FPwatch provides a wealth of information on SDPs in Kinshasa, including specific brands of contraceptives, manufacturers and market prices. Yet FPwatch does not have a parallel population-based survey, nor will be it repeated annually, as is the case for PMA2020. Although it goes beyond the scope of this article, it will be useful to validate key findings from PMA2020 with data from FPwatch.

SDP surveys have an important role in benchmarking facility performance. Regular, representative surveys of facilities provide critical information for benchmarking the performance of selected facilities, such as these PMA sites in Kinshasa. While information on the demand-side of contraceptive use is relatively common in environments like DRC, supply-side data are generally lacking—although critical for a complete understanding of contraceptive use and choice.

To conclude, the SDP module of the PMA2020 survey provides timely, actionable information to the DRC government, FP implementing organizations and donors involved in FP service delivery in Kinshasa, DRC. Yet the value of this information will be determined by the ability of the local FP stakeholders to use it in bringing the needed improvements identified by this survey.

## Funding

PMA2020 is a survey research project implemented by the Bill and Melinda Gates Institute for Population and Reproductive Health at the Johns Hopkins Bloomberg School of Public Health, funded by the Bill and Melinda Gates Foundation (grant # OPP1079004). This article was prepared with support from the Bill and Melinda Gates Foundation (grant 45 #OPP1117997) to the Tulane School of Public Health and Tropical Medicine. The findings and conclusions contained within are those of the authors and do not necessarily reflect positions or policies of the Bill & Melinda Gates Foundation. This study received human subjects approval from Tulane SPHTM (Ref #492318) and from the Kinshasa School of Public Health (2013–2014: ESP/CE/070/13; 2015–2016: ESP/CE/070b/2015; 2015–2016: ESP/CE/070c/2015).

*Conflict of interest statement*. None declared.
